# Enantiomerically Enriched 1,2-*P*,*N*-Bidentate Ferrocenyl Ligands for 1,3-Dipolar Cycloaddition and Transfer Hydrogenation Reactions

**DOI:** 10.3390/molecules23061311

**Published:** 2018-05-30

**Authors:** Irina A. Utepova, Polina O. Serebrennikova, Marina S. Streltsova, Alexandra A. Musikhina, Tatiana G. Fedorchenko, Oleg N. Chupakhin, Andrey P. Antonchick

**Affiliations:** 1 Ural Federal University, 19 Mira Street, Ekaterinburg 620002, Russia; i.a.utepova@urfu.ru (I.A.U.); spo-1803@yandex.ru (P.O.S.); strelcova-marina@mail.ru (M.S.S.); alexa5330@yandex.ru (A.A.M.); 2 Postovsky Institute of Organic Synthesis, Ural Branch of the Russian Academy of Sciences, 22 S. Kovalevskaya Street, Ekaterinburg 620041, Russia; deryabina@ios.uran.ru; 3 Chemische Biologie, Max-Planck-Institut für Molekulare Physiologie, Otto-Hahn Strasse 11, 44227 Dortmund, Germany; andrey.antonchick@mpi-dortmund.mpg.de; 4 Fakultät Chemie und Chemische Biologie, Technische Universität Dortmund, Otto-Hahn-Strasse 4a, 44227 Dortmund, Germany; 5 Faculty of Science, Peoples’ Friendship University of Russia, 6 Miklukho-Maklaya Street, Moscow 117198, Russia

**Keywords:** ferrocenyl ligands, catalysis, cycloaddition, hydrogen transfer

## Abstract

Novel complexes of 1,2-*P*,*N*-bidentate ferrocenyl ligands with AgOAc or with [RuCl_2_(PPh_3_)_3_] as catalysts have been studied in asymmetric synthesis. The catalytic activity of these systems have been studied in [3+2]-cycloaddition of azomethine ylides with olefins and the asymmetric transfer hydrogenation of ketones.

## 1. Introduction

Planar chiral 1,2-bidentate ferrocenyl ligands, as well as binaphthyls, bi(hetero)aryls, and spirobiindans, are widely spread in versatile reactions of asymmetric synthesis reactions [1]. They are connected with the ability, at low concentrations, of the catalyst to provide a high degree of enantioselectivity and high yield of target products. Such ligands are essential for the production of pharmaceuticals and natural products.

To date, a variety of complexes from the available chiral ligands, including ferrocenes, with transition metals have been developed for asymmetric cycloaddition reactions. For example, Cu^I^ or Ag^I^/TF-BiphamPhos (ee up to 99%, C. -J. Wang) [2,3,4,5], Cu^I^/Fesulphos (ee up to 96%, J. C. Carretero) [6,7], Ag^I^/xylyl-FAP (ee up to 97%, X. Zhang) [8], Cu^I^/Ming-Phos (ee up to 95%, J. Zhang) [9], Ag^I^/QUINAP (ee up to 96%, S. L. Schreber) [10], Ag^I^/FPOX (ee up to 98%, Y. -G. Zhou) [11,12,13] and Ag^I^/BINAP (ee up to 99%, J. M. Sansano) [14,15,16,17]. Ferrocene-based catalysts were successfully applied in the asymmetric [3+2]-cycloaddition of azomethine ylides with olefins as well [18,19,20,21,22,23,24,25,26,27]. Moreover, the use of ferrocenyl chiral complexes in small amounts, such as Rh/JoSPOphos [28], Ru/Josiphos [29], and Re^V^/(*R*,*S*)-Josiphos [30], in enantioselective hydrogenation of carbonyl compounds, is significant for chemistry.

**Figure 1:**
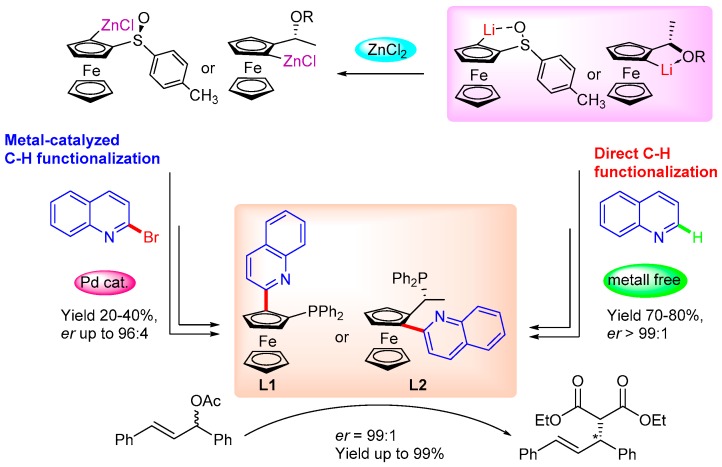
Planar chiral ferrocenyl ligands.

In our previous work, an efficient and straightforward route for the synthesis of planar chiral (hetaryl)ferrocenes *P*,*N*-ligands **L1** (*er* > 99:1) and **L2** (*er* > 99:1) (Figure 1) has been demonstrated [31]. The obtained (hetaryl)ferrocenes revealed high catalytic activity in the Pd-catalyzed Tsuji–Trost allylic substitution [31]. In the present paper, the catalytic activity of chiral complexes based on the ligands **L1** and **L2** in the [3+2]-cycloaddition of azomethine ylides with olefins and the asymmetric transfer hydrogenation of ketones is demonstrated.

## 2. Results and Discussion

**Figure 2:**
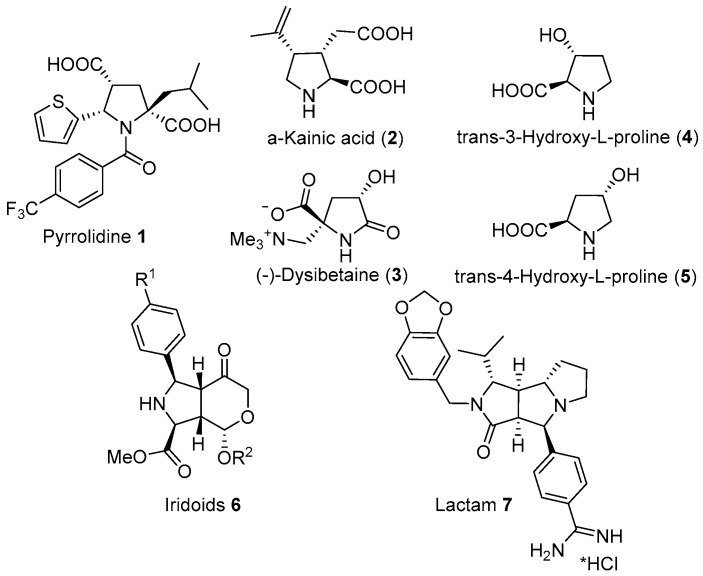
Bioactive and natural compounds based on the pyrrolidine scaffold.

Asymmetric [3+2]-cycloaddition [32] represents one of the most efficient, straightforward, and atom-economical methods for the construction of optically active pyrrolidine derivatives with multiple stereocenters. After pioneering studies of asymmetric pyrrolidines synthesis [8,33], many efforts were directed to the development of synthetic approaches to novel chiral catalysts. Chiral pyrrolidines are present in many biologically active [34,35,36] and natural [37] compounds, and were recently often applied as organocatalysts [38,39,40,41,42,43,44]. For instance compound **1** demonstrates an antiviral activity against hepatitis C (as an inhibitor of RNA polymerase) [45,46], and *α*-kainic acid (**2**) has a neuroexcitatory effect [47]. (–)-Dysibetaine (**3**) is a neuroexcitotoxin which may bind to the glutamate receptors presented in the CNS of mice [48]. Hydroxyprolines **4** and **5** play an important role in the catabolism of collagen, and in the stabilization of protocollagens and glycoproteins in living systems [49]. Derivatives of pyrrolopyrans **6** [23] are known to be the analogues of natural monoterpenoids of the class of cyclopentanopyranes (iridoids), performing protective functions in the organism. Lactam **7** is thrombin inhibitor (Figure 2) [50].

Given the importance of the pyrrolidine scaffold, we studied asymmetric [3+2]-cycloaddition using the developed chiral ligands. Initially, the cycloaddition of methyl (*E*)-2-((4-bromobenzylidene)imino)acetate (**8a**) to *N*-methylmaleimide (**9a**) was selected as a model reaction for testing the activity of new ligands, such as (*S_Fc_*)-[2-(2-quinolin-2-yl)-ferrocen-1-yl]-diphenylphosphine (**L1**) and (*R_Fc_*)-1-(quinolin-2-yl)-2-(*α*-(*R*)-diphenylphosphinoethyl)ferrocene (**L2**). The reaction was carried out in the presence of substoichiometric amounts of triethylamine and tetrakis(acetonitrile)copper(I) tetrafluoroborate (5 mol %) at room temperature. Product **10a** was obtained in 65% yield and *er* = 50:50 in absence of chiral ligand (Table 1, entry 1). Applying complex **L1** with tetrakis(acetonitrile)copper(I) tetrafluoroborate, product **10a** was obtained with an *er* = 76:24 and 45% yield (Table 1, entry 2). Afterwards, we tried to increase the yield and the selectivity of the reaction by employing various Lewis acids, such as AgF and AgOAc with **L1** (Table 1, entries 3 and 4) [2,3,4,15,16,51,52]. The reaction with **L1**/AgF resulted in 58% yield of product **10a** and *er* = 50:50. Using AgOAc with ligand (**L1**), the target product was formed with *er* = 85:15 and 50% yield. Complex **L1** with AgOAc was prepared in situ by stirring for 15 min at room temperature in dichloromethane. There was a change in the color of the reaction mixture from orange to dark red after the formation of the complex. The structure of complex **L1**/AgOAc was confirmed by nuclear magnetic resonance (NMR) spectroscopy, high-resolution mass spectrometry (HRMS), and elemental analysis (see Supplementary Materials page S2, S19, figures S2, S4, S20, S22). The signals of the protons of the ferrocene and heterocyclic moieties of the complex **L1**/AgOAc (3.9–5.4 and 7.4–7.9 ppm) are downfield shifted by 0.1–0.3 ppm and 0.1–0.2 ppm compared to those of the initial **L1** ligand (3.8–5.1 and 7.2–8.0 ppm). In the ^31^P spectra of the complex, a downfield shift by 5.9 ppm relative to the free ligand was also observed.

**Scheme 1:**
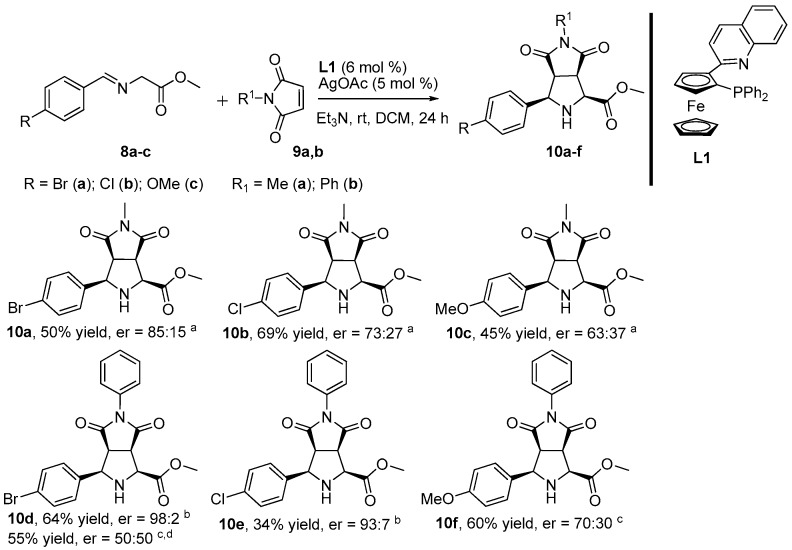
Reaction of [3+2]-cycloaddition of methyl (*E*)-2-((4-substituted-benzylidene)imino)acetate (**8**) and *N*-alkylmaleimide (**9**) catalyzed by the complex **L1**/AgOAc: ^a^ The enantiomeric ratio was determined by high-performance liquid chromatography (HPLC) on a Chiralpak AD. ^b^ The enantiomeric ratio was determined by HPLC on a Chiralcel OD-H. ^c^ The enantiomeric ratio was determined by supercritical fluid chromatography (SFC) on a Chiralcel OD-H. ^d^ Use of the **L2**/AgOAc complex.

Based on the above results, we concluded that the optimal conditions for this transformation are as follows: dichloromethane, 5 mol % AgOAc, 6 mol % ligand, and room temperature. In order to investigate the effect of the substituent on the yield and selectivity of the products, imino esters **8** with donor and acceptor substituents in the benzene ring were synthesized [23,53]. The cycloaddition of methyl (*E*)-2-((4-bromobenzylidene)imino)acetate (**8a**) and methyl (*E*)-2-((4-chlorobenzylidene)imino)acetate (**8b**) with *N*-methylmaleimide (**9a**) led to the formation of products **10a** and **10b** in 50% and 69% yields, respectively (Scheme 1). A similar reaction of **8a** and **8b** with *N*-phenylmaleimide (**9b**) proceeded with the formation of products **10d** and **10e** in 64% and 34% yields and 98:2 and 93:7 *er*, respectively (Scheme 1). The attempt to employ methyl (*E*)-2-((4-methoxybenzylidene)imino)acetate failed. Product **10c** was obtained in 45% yield and *er* = 63:37 using *N*-methylmaleimide as a dipolarophile, and its structural analogue **10f** was obtained in 60% yield and *er* = 70:30 using *N*-phenylmaleimide.

Notably, using (*R*_Fc_)-1-(quinolin-2-yl)-2-(*α*-(*R*)-diphenylphosphinoethyl)ferrocene **L2**, the product **10d** was obtained in 55% yield as a racemate (*er* = 50:50) using the optimized reaction conditions (Scheme 1).

**Scheme 2:**
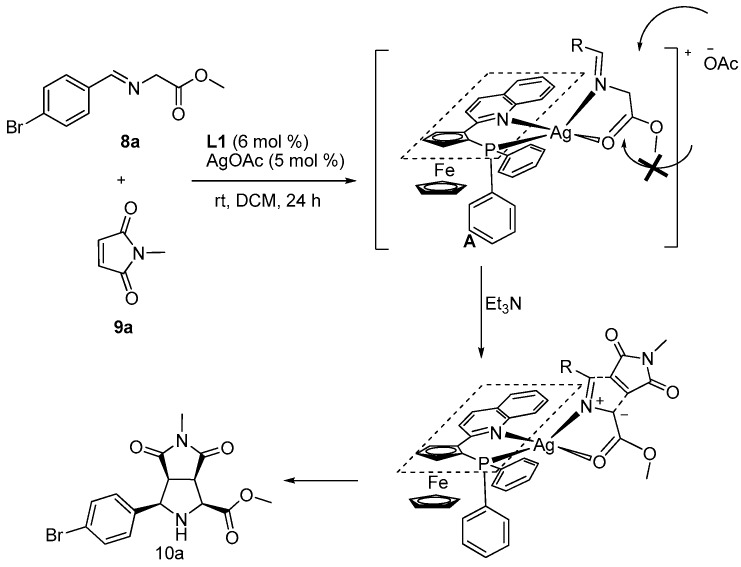
A plausible mechanism of [3+2] cycloaddition of imino ester **8a** and dienophile **9a**.

A plausible mechanism is shown in Scheme 2. In the first step of the reaction, silver(I) is simultaneously coordinated by the bidentate chiral ligand **L1** and the substrate **8** in a tetrahedral arrangement to form the catalytic complex **A**. Next step of deprotonation of complex **A** by triethylamine leads to the formation of the azomethine ylide, the active substrate for the cycloaddition. This active substrate then undergoes a cycloaddition with dienophile **9** to furnish the product **10a** (Scheme 2). The dienophile attacks from the less-hindered side (above the plane), to avoid unfavorable steric interactions with the bulky diphenylphosphine group of the ligand [20].

**Figure 3:**
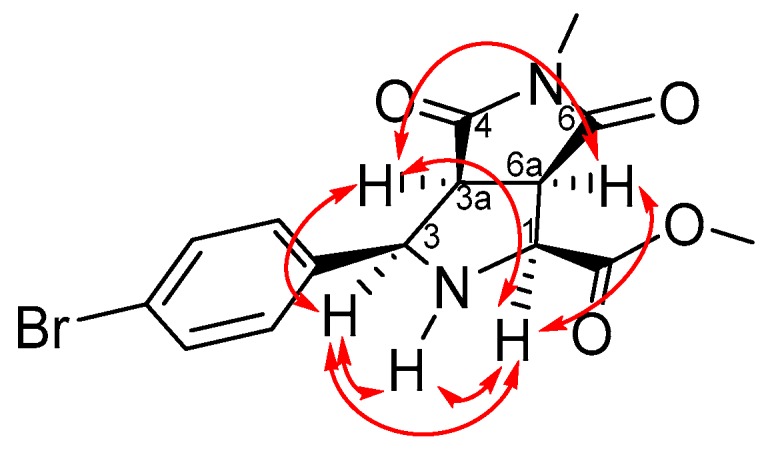
NOESY (H↔H) correlations of compound **10a**.

The structures of the cycloaddition products **10a**–**f** have been confirmed by NMR spectroscopy, and the data corresponding well with the literature [53,54]. In addition, we carried out nuclear Overhauser effect spectroscopy (NOESY) experiments for compound **10a**, to gain structural and stereochemical information. The NOESY experiment showed a NOE effect of the H-3 to H-3a, and H-1 to H-6a, enabling the determination of the stereochemistry of this compound as *endo*-product (Figure 3). Absolute configuration was determined by comparison with the literature data [54]. The enantiomeric ratio of the compounds was determined by high-performance liquid chromatography (HPLC) and supercritical fluid chromatography (SFC) analysis.

**Figure 4:**
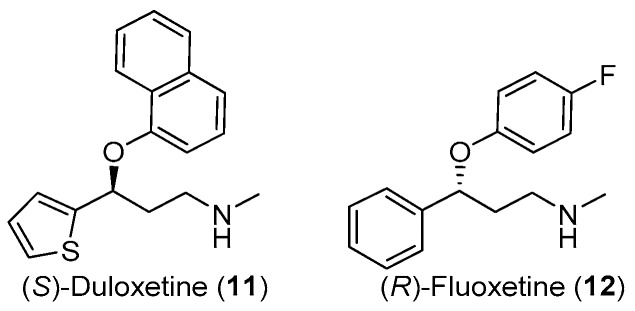
Biologically active compounds obtained by the reaction of asymmetric reduction.

The asymmetric hydrogenation of double bonds is known to be of great importance in the synthesis of biologically active compounds and their precursors. For example, (*S*)-Duloxetine (**11**) is an antidepressant drug targeting the presynaptic cell and (*R*)-Fluoxetine (**12**) is an antidepressant of the selective serotonin reuptake inhibitor (SSRI) class (Figure 4) [55,56,57]. In particular, ferrocenyl chiral complexes [29,30,58,59] are utilized for the carbonyl group reduction.

**Scheme 3:**
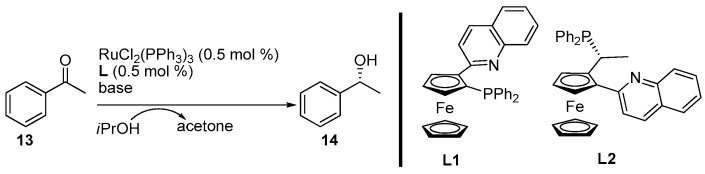
Asymmetric transfer hydrogenation of acetophenone **13** in the presence of the complex **L1**/RuCl_2_(PPh_3_)_3_ or **L2**/RuCl_2_(PPh_3_)_3_.

Next, we tried to examine the catalytic activity of planar chiral ferrocenyl ligands **L1** and **L2** applying the Ru-catalyzed transfer hydrogenation of ketones with acetophenone **13** as a model substrate (Scheme 3, Table 2).

According to the standard procedure [29], the reaction was carried out in the presence of catalytic amounts of base and the active complex **L1**/RuCl_2_(PPh_3_)_3_ in degassed *i*PrOH. The reaction of **L1** with [RuCl_2_(PPh_3_)_3_] in toluene at room temperature for 10 min gave the desired red complex. The structure of complex **L1**/RuCl_2_(PPh_3_)_3_ has been confirmed by NMR spectroscopy HRMS and elemental analysis (see Supplementary Materials page S2, S22, figures S3, S5, S21, S23). The ^1^H-NMR spectra point out that the proton signals of the ferrocene and heterocyclic moieties of the complex **L1**/RuCl_2_(PPh_3_)_3_ (3.9–5.4 and 7.4–7.9 ppm) in comparison to the initial ligand **L1** (3.8–5.1 and 7.2–8.0 ppm) are downfield shifted by 0.1–0.3 ppm and 0.1–0.2 ppm, respectively. It should be noted that in the ^31^P spectra of the complex, the signal is downfield shifted by 5.6 ppm relative to the initial ligand. 

Asymmetric transfer hydrogenation of acetophenone **13** to (*R*)-1-phenylethanol **14** was carried out in the presence of 0.5 mol % of complex **L1**/RuCl_2_(PPh_3_)_3_ and 2 mol % *t*-BuOK. The reaction mixture was stirred under argon atmosphere for 20 h at room temperature. (*R*)-1-phenylethanol **14** was isolated by column chromatography on SiO_2_ in 20% yield and with the selectivity of more than 99% (Table 2, entry 2). The asymmetric transfer hydrogenation conditions were optimized with respect to the type and amount of base and the reaction temperature (Table 2).

The application of 4-dimethylaminopyridine (DMAP) and NaH as bases provided low yields of product **14** (Table 2, entries 3 and 4). Furthermore, **14** was obtained in 2% yield after 72 h in the presence of 20 mol % using triethylamine as base (Table 2, entry 5). Next, we tried to increase the yield by heating to 80 °C (Table 2, entry 6). At last, (*R*)-1-phenylethanol **14** in 98% yield was achieved by carrying out the reaction at 80 °C in the presence of 20 mol % *t*-BuOK (Table 2, entry 7). Reducing the loading of *t*-BuOK to 10 and 2 mol % resulted in a slight decrease in the product yield (Table 2, entries 8 and 9). The selectivity of the process in all above cases with **L1**/RuCl_2_(PPh_3_)_3_ was more than 99%.

In the reaction with **L2**/RuCl_2_(PPh_3_)_3_ complex, the selectivity of (*R*)-1-phenylethanol (**14**) was also more than 99% with a yield of 53% (Table 2, entry 10).

**Scheme 4:**
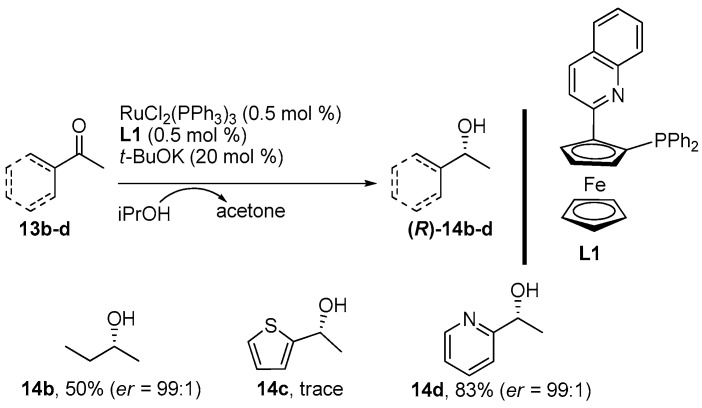
Asymmetric transfer hydrogenation of carbonyl compounds **13** in the presence of the complex **L1**/RuCl_2_(PPh_3_)_3_.

Based on the above results, we conclude that the optimal conditions for this transformation are as follows: 20 mol % *t*-BuOK, 0.5 mol % ligand, 0.5 mol % RuCl_2_(PPh_3_)_3_ and 80 °C. In order to investigate the effect of the substituent on the yield and selectivity of the products, other ketones were used (Scheme 4). The asymmetric transfer hydrogenation of 2-butanone **13b** led to the formation of products **14b** in 50% yields. Next, we investigated the ability of using of asymmetric transfer hydrogenation for π-excess and π-deficient carbonyl compounds such as 2-acetylthiophene **13c** and 2-acetylpyridine **13d** (Scheme 4). (*R*)-1-(Thiophen-2-yl)ethan-1-ol **14c** was isolated in trace amounts. This is interpreted by the instability of thiophene derivatives. As opposed to 2-acetylthiophene, the asymmetric transfer hydrogenation of π-deficient 2-acetylpyridine **13d** led to the formation of products **14d** in high yield and selectivity (Scheme 4).

## 3. Materials and Methods

### 3.1. General Information

All reactions were carried out under argon using standard Schlenk Techniques (Hofmann Glastechnik GmbH, Staudt, Germany). The ^1^H-NMR (400 MHz), ^13^C-NMR (100 MHz), ^31^P-NMR (162 MHz) spectra were recorded on a NMR spectrometer (400 MHz). Chemical shifts are given in *δ* values (ppm) using tetramethylsilane (TMS) as internal standard and CDCl_3_ as solvent. Electrospray mass spectra were recorded in positive mode with maXis impact high resolution Q-TOF mass spectrometer (Bruker Daltonics, Bremen, Germany) in 50–2500 Da mass range by direct infusion of sample solutions in methanol using kdScientific (KD Scientific Inc., Holliston, MA, USA) syringe pump at 120 µL/h flow rate. Modified instrument settings of a pre-installed method Direct_Infusion_100–1000 were used. Mass calibration was performed using ES-TOF G1969-85000 tuning mix (Agilent Technologies) by HPC method (High Precision Calibration standard procedure by Bruker). All data were collected and analyzed with Compass for oTof series 1.7/DataAnalysis 4.2 software package (Bruker, Germany). Analytical HPLC (Knauer, Berlin, Germany) and SFC (Waters Corporation, Milford, MA, USA) were performed using a chiral column. The angle of rotation was measured on a polarimeter Perkin-Elmer 343+ (PerkinElmer Instruments, Llantrisant, Wales, UK). The elemental analysis was carried out on a CHNS/O analyzer Perkin-Elmer (PerkinElmer Instruments, Norwalk, CT, USA). The course of the reactions was monitored by thin layer chromatography (TLC) on silica gel plates (Macherey-Nagel GmbH & Co, Düren, Germany). The column chromatography was performed on silica gel (silica gel 60, 0.035−0.070 mm, 220−440 mesh). 

Glycine methyl ester hydrochloride, *N*-methylmaleimide, *N*-phenylmaleimide, benzaldehydes, acetophenone, Cu(CH_3_CN)_4_BF_4_, AgF, AgOAc, and [RuCl_2_(PPh_3_)_3_] were purchased from Aldrich (Merck KGaA, Darmstadt, Germany). (*S_Fc_*)-[2-(2-quinolin-2-yl)-ferrocen-1-yl]-diphenylphosphine (**L1**), (*R_Fc_*)-1-(quinolin-2-yl)-2-(*α*-(*R*)- diphenyl-phosphinoethyl)ferrocene (**L2**) [31] and imine **8** [23] were prepared according to the published procedures.

### 3.2. General Procedure for the Synthesis of Products ***10***

Ligand **L1** (6 mol %) and AgOAc (5 mol %) were dissolved in dichloromethane (1.0 mL) and the solution was stirred for 15 min at room temperature. Then, dipolarophile **9** (1.2 mmol), imine **8** (1 mmol) and Et_3_N (20 mol %) were added to the mixture and stirred at room temperature for 24 h. After evaporation under reduced pressure, the residue was purified through column chromatography on silica gel (hexane/ethyl acetate = 9/1) to give the pure products. The structures of products were known [53,54] and confirmed by NMR. The enantiomeric excesses of the products were determined by HPLC and SFC on chiral stationary phase.

*Methyl (1R,3S,3aR,6aS)-3-(4-bromophenyl)-5-methyl-4,6-dioxooctahydropyrrolo[3,4-c]pyrrole-1-carboxylate* (**10a**) [54]. Obtained from imine **8a** and *N*-methylmaleimide **9a**, 0.183 g, 50%, white solid; [*α*]_D_^20^ = +2.7 (*c* 0.16, CDCl_3_); HPLC: Chiralpak AD, λ = 220 nm, hexane/*i*PrOH/MeOH, 50:20:5, 0.6 mL/min, retention time (t_R_): 20.5 min and 27.8 min.

*Methyl (1R,3S,3aR,6aS)-3-(4-chlorophenyl)-5-methyl-4,6-dioxooctahydropyrrolo[3,4-c]pyrrole-1-carboxylate* (**10b**) [54]. Obtained from imine **8b** and *N*-methylmaleimide **9a**, 0.222 g, 69%, gray solid; [α]_D_^20^ = +2.4 (*c* 0.15, CDCl_3_); HPLC: Chiralpak AD, λ = 220 nm, hexane/*i*PrOH/MeOH, 50:25:5, 1 mL/min, t_R_: 10.45 min and 14.22 min.

*Methyl(1R,3S,3aR,6aS)-3-(4-methoxyphenyl)-5-methyl-4,6-dioxooctahydropyrrolo[3,4-c]pyrrole-1-carboxylate* (**10c**) [54]. Obtained from imine **8b** and *N*-methylmaleimide **9a**, 0.143 g, 45%, dark beige solid; [α]_D_^20^ = −0.7 (*c* 0.12, CDCl_3_); HPLC: Chiralpak AD, λ = 220 nm, hexane/*i*PrOH/MeOH, 50:25:5, 1 mL/min, t_R_: 13.87 min and 17.42 min.

*Methyl (1R,3S,3aR,6aS)-3-(4-bromophenyl)-4,6-dioxo-5-phenyloctahydropyrrolo[3,4-c]pyrrole-1-carboxylate* (**10d**) [53]. Obtained from imine **8a** and *N*-phenylmaleimide **9b**, 0.274 g, 64%, light gray solid; [α]_D_^20^ = +61.3 (*c* 0.15, CDCl_3_); SFC: Chiralcel OD-H, λ = 220 nm, CO_2_/MeOH, 75:25, 0.8 mL/min, 140 bar, t_R_: 15.74 min and 24.67 min).

*Methyl (1R,3S,3aR,6aS)-3-(4-chlorophenyl)-4,6-dioxo-5-phenyloctahydropyrrolo[3,4-c]pyrrole-1-carboxylate* (**10e**) [53]. Obtained from imine **8b** and *N*-phenylmaleimide **9b**, 0.130 g, 34%, dark gray solid; [α]_D_^20^ = +3.4 (*c* 0.1, CDCl_3_); HPLC: Chiralcel OD-H, λ = 220 nm, hexane/*i*PrOH/MeOH, 50:20:5, 1 mL/min, t_R_: 21.80 min and 28.18 min).

*Methyl(1R,3S,3aR,6aS)-3-(4-methoxyphenyl)-4,6-dioxo-5-phenyloctahydropyrrolo[3,4-c]pyrrole-1-carboxylate* (**10f**) [53]. Obtained from imine **8c** and *N*-phenylmaleimide **9b**, 0.228 g, 60%, beige solid; [α]_D_^20^ = −0.2 (*c* 0.14, CDCl_3_); SFC: Chiralcel OD-H, λ = 220 nm, CO_2_/MeOH, 75:25, 0.8 mL/min, 140 bar, t_R_: 15.57 min and 22.28 min.

### 3.3. General Procedure for the Synthesis of Products ***14***

A solution of ketone (1 mmol) and a base (20 mol %) was added to a solution of complex **L1**/RuCl_2_(PPh_3_)_3_ (0.5 mol %) in degassed *i*PrOH (30 mL). The reaction mixture was stirred at 80 °C for 20 h, and the progress of the reaction was monitored by TLC. After evaporation under reduced pressure, the residue was purified through column chromatography on silica gel to yield pure product. The structures of products were known and were confirmed by NMR spectroscopy.

*(R)-1-Phenylethanol* (**14a**) [30]. Obtained from acetophenone **13a**, 0.120 mg, 98%, yellow oil; [*α*]_D_^20^ = +1.3 (*c* 0.06, CDCl_3_); SFC: Lux Amylose-2, λ = 210 nm, CO_2_/MeOH, 98:2, 1 mL/min, 140 bar, t_R_: 6.1 min.

*(R)-2-Butanol* (**14b**) [60]. Obtained from 2-butanone **13b**, 0.037 g, 50%; SFC: Lux Amylose-2, λ = 275 nm, CO_2_/MeOH, 95:5, 0.8 mL/min, 140 bar, t_R_: 5.1 min.

*(R)-1-(Pyridin-2-yl)ethanol* (**14d**) [61]. Obtained from 2-acetylpyridine **13d**, 0.102 g, 83%; SFC: Chiralcel OD-H, λ = 266 nm, CO_2_/MeOH, 90:10, 1 mL/min, 140 bar, *t*_R_: 2.4 min.

## 4. Conclusions

New Ag- and Ru-catalysts based on the planar chiral ferrocene *P*,*N*-ligands **L1** and **L2** have been obtained. The scope of activity of the complexes was demonstrated in two types of asymmetric reactions: [3+2]-cycloaddition of azomethine ylides with olefins and asymmetric transfer hydrogenation of carbonyl compounds. The catalytic activity of the ligands **L1** and **L2** has been shown to be comparable with the ones of previously described ligands in the presented transformations. Thus, the obtained complexes revealed high reactivity and good enantioselectivity and enabled to access to optically active pyrrolidine derivatives and products of reduction of multiple bonds under mild conditions. As the final step, we succeeded to apply the approach to the synthesis of potential biologically active compounds.

## Figures and Tables

**Table 1:**
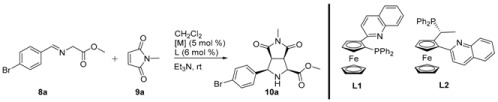
Asymmetric [3+2]-cycloaddition of methyl (*E*)-2-((4-bromobenzylidene)imino)acetate **8a** and *N*-methylmaleimide **9a**.

Entry	Ligand	[M]/Base	Yield (%) 10a	*er* (%) 10a
1	-	[Cu(CH_3_CN)_4_][BF_4_]/Et_3_N	65	50:50
2	**L1**	[Cu(CH_3_CN)_4_][BF_4_]/Et_3_N	45	76:24
3	**L1**	AgF/Et_3_N	50	50:50
4	**L1**	AgOAc/Et_3_N	50	85:15

**Table 2:** Transfer hydrogenation of acetophenone **15** under different reaction conditions.

Entry	Ligand	*T* (°C)	Base (mol %)	Time (h)	Yield (%) 14	*er* (%) ^1^ 14
1	**-**	RT	*t*-BuOK (2)	48	30 ^2^	1:1
2	**L1**	RT	*t*-BuOK (2)	48	20 ^2^	>99:1
3	**L1**	RT	DMAP (2)	48	-	-
4	**L1**	RT	NaH (2)	48	11 ^3^	>99:1
5	**L1**	RT	Et_3_N (20)	72	2 ^3^	>99:1
6	**L1**	80	Et_3_N (20)	20	40 ^3^	>99:1
7	**L1**	80	*t*-BuOK (20)	20	98 ^3^	>99:1
8	**L1**	80	*t*-BuOK (10)	20	97 ^3^	>99:1
9	**L1**	80	*t*-BuOK (2)	20	96 ^3^	>99:1
10	**L2**	80	*t*-BuOK (20)	20	53 ^3^	>99:1

^1^ The enantiomeric ratio was determined by SFC on a Lux Amylose-2. ^2^ The product was isolated by column chromatography on SiO_2_. ^3^ The yield was determined by NMR spectroscopy.

## Supplementary Materials

The following are available online. The Supporting Materials contains copies of all ^1^H-, ^13^C- and ^31^P-NMR spectra, HPLC, SFC, HRMS and elemental analysis data.
