# Supplementary material for: Enantiomerically Enriched 1,2-P,N-Bidentate Ferrocenyl Ligands for 1,3-Dipolar Cycloaddition and Transfer Hydrogenation Reactions
Source: Molecules. 2018 May 30;23(6):1311. doi: 10.3390/molecules23061311 (PMC6100496; doi:10.3390/molecules23061311)
Supplement: Supplementary file 1 [file molecules-23-01311-s001.zip › molecules-309980-supplementary.doc]

*Supplementary Material:*

Enantiomerically Enriched 1,2‐*P*,*N*‐Bidentate Ferrocenyl Ligands for 1,3-Dipolar Cycloaddition and Transfer Hydrogenation Reactions

Irina A. Utepova ^1,2^, Polina O. Serebrennikova ^1^, Marina S. Streltsova ^1^, Alexandra A. Musikhina ^1^, Tatiana G. Fedorchenko ^2^, Oleg N. Chupakhin ^1,2,^* and Andrey P. Antonchick ^3,4,5^

^1^	Ural Federal University, 19 Mira Street, Ekaterinburg 620002, Russia; i.a.utepova@urfu.ru (I.A.U.); spo-1803@yandex.ru (P.O.S.); strelcova-marina@mail.ru (M.S.S.); alexa5330@yandex.ru (A.A.M.)

^2^	Postovsky Institute of Organic Synthesis, Ural Branch of the Russian Academy of Sciences,
22 S. Kovalevskaya Street, Ekaterinburg 620041, Russia; deryabina@ios.uran.ru

^3^	Chemische Biologie, Max-Planck-Institut für Molekulare Physiologie, Otto-Hahn Strasse 11,
44227 Dortmund, Germany; andrey.antonchick@mpi-dortmund.mpg.de

^4^	Fakultät Chemie und Chemische Biologie, Technische Universität Dortmund, Otto-Hahn-Strasse 4a,
44227 Dortmund, Germany

^5^	Faculty of Science, Peoples’ Friendship University of Russia, 6 Miklukho-Maklaya Street,
Moscow 117198, Russia

*****	Correspondence: chupakhin@ios.uran.ru; Tel.: +7-343-374-1189

Table of Contents

Experimental section.	S2

^1^H NMR Spectra	S4

^13^C NMR Spectra	S10

^31^P NMR Spectra	S13

NOESY Spectra	S16

HPLC and SFC analysis data	S17

HRMS data	S22

**Experimental section**

A solution of freshly prepared ligand (0.034 g, 0.07 mmol) in CH_2_Cl_2_ (2 mL) was added with stirring to a solution of AgOAc (0.012 g, 0.07 mmol) in CH_2_Cl_2_ (2 mL). The reaction mixture was stirred at room temperature for 15 min. The red solution was concentrated under reduce pressure. The residue was dissolved in hexane and filtered off from the unreacted AgOAc. Solution was concentrated under reduce pressure. The solid was treated by ethyl ether. The brown solids were separated from the solution by filtration and washed with Et_2_O. Found (%): C, 59.21; H, 3.83; N, 2.55. C_33_H_27_AgFeNO_2_P. Calculated (%): C, 59.67; H, 4.07; N, 2.11. ESI-MS, m/z (%): [M+L]^+^ = 1102.1008 (9.4%). The structure of complex **L1**/AgOAc was confirmed by NMR spectroscopy (Figure S2), HRMS (Page 21) and elemental analysis. The signals of the protons of the ferrocene and heterocyclic moieties of the complex **L1**/AgOAc (3.9-5.4 and 7.4-7.9 ppm) are downfield shifted by 0.1-0.3 ppm and 0.1-0.2 ppm compared to those of the initial **L1** ligand (3.8-5.1 and 7.2-8.0 ppm) (Figure S4). In the ^31^P spectra of the complex, a downfield shift by 5.9 ppm relative to the initial compound was also observed (Figure S22).

A solution of freshly prepared ligand (0.015 g, 0.03 mmol) in toluene (5 mL) was added with stirring to a suspension of RuCl_2_(PPh_3_)_3_ (0.03 g, 0.03 mmol) in toluene (3 mL). The reaction mixture was stirred at room temperature for 15 min. The dark brown solution was concentrated under reduce pressure. The solid was treated by 50mL Et_2_O. The dark brown solids were separated from the solution by filtration and washed with Et_2_O. Found (%): C, 63.63; H, 3.84; N, 1.37. C_49_H_39_Cl_2_FeNP_2_Ru. Calculated (%): C, 63.16; H, 4.19; N, 1.50. ESI-MS, m/z (%): [M-PPh_3_]^+^ = 669.2323 (0.2%). The structure of complex **L1**/[RuCl_2_(PPh_3_)_3_] has been confirmed by NMR spectroscopy (Figure S3), HRMS (Page 24) and elemental analysis. The ^1^H NMR spectra point out that the proton signals of the ferrocene and heterocyclic moieties of the complex **L1**/RuCl_2_(PPh_3_)_3_ (3.9-5.4 and 7.4-7.9 ppm) in comparison to the initial ligand **L1** (3.8-5.1 and 7.2-8.0 ppm) are downfield shifted by 0.1-0.3 ppm and 0.1-0.2 ppm, respectively (Figure S5). It should be noted, that in the ^31^P spectra of the complex, the signal is downfield shifted by 5.6 ppm (Figure S4) relative to the initial ligand.

**^1^H NMR Spectra**

**Figure S1.** ^1^H NMR spectrum for (*S_Fc_*)-[2-(2-quinolin-2-yl)-ferrocen-1-yl]-diphenylphosphine L1

**Figure S2.** ^1^H NMR spectrum for complex L1/AgOAc

**Figure S3.** ^1^H NMR spectrum for complex L1/RuCl_2_(PPh_3_)_3_

**Figure S4.** ^1^H NMR spectrum for L1*BH_3_, L1 and L1/AgOAc

**Figure S5**. ^1^H NMR spectrum for L1*BH_3_, L1 and L1/RuCl_2_(PPh_3_)_3_

**Figure S6.** ^1^H NMR spectrum for methyl (*1R*,*3S*,*3aR*,*6aS*)-3-(4-bromophenyl)-5-methyl-4,6-dioxooctahydropyrrolo[3,4-*c*]pyrrole-1-carboxylate 10a

**Figure S7**. ^1^H NMR spectrum for methyl (*1R*,*3S*,*3aR*,*6aS*)-3-(4-chlorophenyl)-5-methyl-4,6-dioxooctahydropyrrolo[3,4-*c*]pyrrole-1-carboxylate 10b

**Figure S8**. ^1^H NMR spectrum for methyl (*1R*,*3S*,*3aR*,*6aS*)-3-(4-methoxyphenyl)-5-methyl-4,6-dioxooctahydropyrrolo[3,4-*c*]pyrrole-1-carboxylate 10c

**Figure S9**. ^1^H NMR spectrum for methyl (*1R*,*3S*,*3aR*,*6aS*)-3-(4-bromophenyl)-4,6-dioxo-5-phenyloctahydropyrrolo[3,4-*c*]pyrrole-1-carboxylate 10d

**Figure S10**. ^1^H NMR spectrum for methyl (*1R*,*3S*,*3aR*,*6aS*)-3-(4-chlorophenyl)-4,6-dioxo-5-phenyloctahydropyrrolo[3,4-*c*]pyrrole-1-carboxylate 10e

**Figure S11**. ^1^H NMR spectrum for methyl (*1R*,*3S*,*3aR*,*6aS*)-3-(4-methoxyphenyl)-4,6-dioxo-5-phenyloctahydropyrrolo[3,4-*c*]pyrrole-1-carboxylate 10f

**Figure S12**. ^1^H NMR spectrum for (*R*)-1-phenylethanol 14

**^13^C NMR Spectra**

**Figure S13.** ^13^C NMR spectrum for methyl (*1R*,*3S*,*3aR*,*6aS*)-3-(4-bromophenyl)-5-methyl-4,6-dioxooctahydropyrrolo[3,4-*c*]pyrrole-1-carboxylate 10a

**Figure S14**. ^13^C NMR spectrum for methyl (*1R*,*3S*,*3aR*,*6aS*)-3-(4-chlorophenyl)-5-methyl-4,6-dioxooctahydropyrrolo[3,4-*c*]pyrrole-1-carboxylate 10b

**Figure S15.** ^13^C NMR spectrum for methyl (*1R*,*3S*,*3aR*,*6aS*)-3-(4-methoxyphenyl)-5-methyl-4,6-dioxooctahydropyrrolo[3,4-*c*]pyrrole-1-carboxylate 10c

**Figure S16.** ^13^C NMR spectrum for methyl (*1R*,*3S*,*3aR*,*6aS*)-3-(4-bromophenyl)-4,6-dioxo-5-phenyloctahydropyrrolo[3,4-*c*]pyrrole-1-carboxylate 10d

**Figure S17.** ^13^C NMR spectrum for methyl (*1R*,*3S*,*3aR*,*6aS*)-3-(4-chlorophenyl)-4,6-dioxo-5-phenyloctahydropyrrolo[3,4-*c*]pyrrole-1-carboxylate 10e

**Figure S18.** ^13^C NMR spectrum for methyl (*1R*,*3S*,*3aR*,*6aS*)-3-(4-methoxyphenyl)-4,6-dioxo-5-phenyloctahydropyrrolo[3,4-*c*]pyrrole-1-carboxylate 10f

**^31^P NMR Spectrum**

**Figure S19**. ^31^P NMR spectrum for (*S_Fc_*)-[2-(2-quinolin-2-yl)-ferrocen-1-yl]-diphenylphosphine L1

**Figure S20.** ^31^P NMR spectrum for L1/AgOAc complex

**Figure S21**. ^31^P NMR spectrum for L1/RuCl_2_(PPh_3_)_3_ complex

**Figure S22**. ^31^P NMR spectrum for L1*BH_3_, L1 and L1/AgOAc

**Figure S23**. ^31^P NMR spectrum for L1*BH_3_, L1 and L1/RuCl_2_(PPh_3_)_3_

**NOESY Spectra**

**Figure S24**. NOESY (H↔H) correlations of methyl (*1R*,*3S*,*3aR*,*6aS*)-3-(4-bromophenyl)-5-methyl-4,6-dioxooctahydropyrrolo[3,4-*c*]pyrrole-1-carboxylate 10a

**HPLC and SFC analysis data**

**Figure S25**. HPLC data for methyl (*1R*,*3S*,*3aR*,*6aS*)-3-(4-bromophenyl)-5-methyl-4,6-dioxooctahydropyrrolo[3,4-*c*]pyrrole-1-carboxylate 10a which was obtained without the use of a L1

**Figure S26**. HPLC data for methyl (*1R*,*3S*,*3aR*,*6aS*)-3-(4-bromophenyl)-5-methyl-4,6-dioxooctahydropyrrolo[3,4-*c*]pyrrole-1-carboxylate 10a

**Figure S27.** HPLC data for methyl (*1R*,*3S*,*3aR*,*6aS*)-3-(4-chlorophenyl)-5-methyl-4,6-dioxooctahydropyrrolo[3,4-*c*]pyrrole-1-carboxylate 10b

**Figure S28**. HPLC data for methyl (*1R*,*3S*,*3aR*,*6aS*)-3-(4-methoxyphenyl)-5-methyl-4,6-dioxooctahydropyrrolo[3,4-*c*]pyrrole-1-carboxylate 10c

**Figure S29**. SFC data for methyl (*1R*,*3S*,*3aR*,*6aS*)-3-(4-bromophenyl)-4,6-dioxo-5-phenyloctahydropyrrolo[3,4-*c*]pyrrole-1-carboxylate 10d

**Figure S30.** HPLC data for methyl (*1R*,*3S*,*3aR*,*6aS*)-3-(4-chlorophenyl)-4,6-dioxo-5-phenyloctahydropyrrolo[3,4-*c*]pyrrole-1-carboxylate 10e

**Figure S31**. SFC data for methyl (*1R*,*3S*,*3aR*,*6aS*)-3-(4-methoxyphenyl)-4,6-dioxo-5-phenyloctahydropyrrolo[3,4-*c*]pyrrole-1-carboxylate 10f

**Figure S32.** SFC data for (*R*)-1-phenylethanol 14

**HRMS data**

1. HRMS data for complex **L1**/AgOAc

2. HRMS data for complex **L1**/RuCl_2_(PPh_3_)_3_
